# Anterior-interrupted and posterior-continuous suture technique improved the success rate of kidney transplantation model in rats

**DOI:** 10.1590/acb396024

**Published:** 2024-09-20

**Authors:** Lan-Tao Lu, Xun-Feng Zou, Shuang-Qing Han

**Affiliations:** 1 Fifth Tianjin Central Hospital – Department of General Surgery – Tianjin – China.; 2 Nankai University – Department of General Surgery – First Central Hospital – Tianjin – China.; 3 Tianjin Medical University – Central Hospital Clinic Institute – Tianji – China.

**Keywords:** Anastomoses, Surgical, Kidney Transplantation, Models, Animal, Suture Techniques

## Abstract

**Purpose:**

This study aimed to introduce and evaluate two new microvascular anastomosis techniques compared to the conventional method in a rat renal transplant model.

**Methods:**

Using a Fisher-to-Lewis rat kidney transplantation model, the renal artery anastomosis was performed using the interrupted (I) suture technique, Y-shaped continuous (Y) suture technique, and anterior-interrupted and posterior-continuous (I-C) suture technique. The rats were then divided into three groups: I group, Y group, and I-C group. Parameters such as arterial anastomosis time, warm ischemia time, seven-day survival rate of the rats, and vessel histopathology were assessed.

**Results:**

The mean arterial anastomosis time, blood leakage scores, and warm ischemia time were significantly reduced in groups Y and I-C compared to group I. Moreover, the seven-day survival rate was significantly higher in the I-C group compared to the other two groups. Arterial histopathology demonstrated vessel wall recovery without damage in all three groups, suggesting the safety of both Y and I-C techniques.

**Conclusions:**

The anterior-interrupted and posterior-continuous suture method is particularly beneficial for small artery reconstruction in organ transplantation.

## Introduction

Renal artery anastomosis, in which arteries are small with external diameter ranging from 0.5 to 0.8 mm, is a crucial step in the rat kidney transplantation model, with main challenges including anastomotic bleeding, vascular obstruction, and prolonged warm ischemia caused by lengthy suture time^1^. To ensure success, the warm ischemia time of the donor kidney must be strictly limited to 30 minutes. Prolonged warm ischemia can lead to higher rates of acute tubular necrosis and lower renal graft survival. It is vital for organ function restoration in renal transplantation model by minimizing the time for vascular anastomosis. The interrupted (I) suture technique^2^ is normally used in arterial anastomosis, but it is time-consuming and carries a high risk of bleeding, hindering renal transplant performance. Therefore, microsurgeons aim to enhance arterial suture methods to expedite vessel anastomosis stability, ultimately improving the success rate of the renal transplantation model.

The continuous suturing technique in blood vessel operations is known for its efficiency in reducing excessive knotting^3^. Studies have shown that this method can be a viable alternative to traditional interrupted sutures, with similar vessel patency outcomes^4^. However, when anastomosing small arteries, there is a heightened risk of vascular lumen narrowing due to the pocket effect at the anastomosis site. To mitigate this risk, the Y-shaped (Y) suture technique involves continuously suturing the anterior and posterior walls of the arterial anastomosis.

Rat kidney transplantation serves as a crucial model for investigating immune rejection, immune tolerance, and transplant-related diseases. A key factor affecting the success of orthotopic kidney transplantation in this model is the renal artery anastomosis technique. In order to combine the advantages of continuous sutures with the potential of interrupted sutures^5^, our group adopted an anterior-interrupted and posterior-continuous (I-C) suture technique, which was used to anastomose the renal arteries of rats.

Thus, this study aimed to assess the feasibility of these two suture techniques within the model and compare them with the traditional I suture technique. The study evaluated their impact on the survival rate and renal function of transplanted kidneys following allogeneic kidney transplantation.

## Methods

Male inbred Fisher (F344, RT11^vr^) rats and Lewis (LEW, RT^1^) rats, weighing 250 to 300 g, were used as donor and recipients, respectively. Animals were housed in 12:12-hour light/dark cycle and allowed free access to water and standard rat chow. Animal protocols were approved by the institutional review boards. Animals were anesthetized with sodium pentobarbital (50 mg/kg, intraperitoneal). The studies met the Nankai University Research Council’s guide for the care and use of laboratory animals in China.

For suture techniques, 10/0-nylon suture was utilized. A Y-shape suture was made by tying two 10/0-nylon sutures together in the middle and cutting off one of their non-needle ends. An operation microscope with 16 to 20× magnification (Stemi, 2000-C, Carl Zeiss MicroImagin) and an electro-coagulator (ICC 50, ERBE Elektromedizin) were used. Isoflurane inhalation was used for anesthesia. The concentration of isoflurane was 2%, and the oxygen flow was 0.5 L per minute.

### Kidney transplantation model in rats and experimental design

Orthotopic renal transplantation was performed according to a previous report^6^.

The donor operation consisted of the procurement of the donor kidney involved injecting 2-mL saline with 200 uL of heparin solution into the dorsal vein of the penis to achieve systemic heparinization. The donor rat was anesthetized intraperitoneally. Following an abdominal incision along the midline, the abdominal cavity was opened, and the large and small bowels were covered with saline-soaked gauze, then retracted to expose the left kidney in the right abdomen. The renal vein branches were dissociated and ligated with a 5/0-silk tie. Dissection was performed on the left renal vessels and part of the aorta around the renal artery to expose the aorta. The abdominal aorta was blocked above and below the renal artery by vascular forceps, and a 22G venous catheter was inserted into the lumbar aorta. The left kidney was flushed with cold (4°C) preservation solution (5 mL/5 min). The left kidney vein was cut at its junction with the inferior vena cava to allow venous flow of the flushing solution. Subsequently, the left renal artery was divided adjacent to the aorta, and the donor kidney was cold stored in the same organ preservation solution until implantation.

For the recipient operation, the preparation and dissection of renal vessels in the donor operation were similar to that of the recipient. The left ureter was dissected at the renal subpolar plane, and two microvascular clamps were applied on the renal artery and vein near their roots. The native left kidney was then removed after division of the distal vessels. The donor kidney was positioned in an orthotopic manner and covered with an ice-cold saline gauze. Arterial anastomosis was performed in an end-to-end fashion using 10/0-silk sutures, through three different suture techniques as per the experimental design.

**Figure 1:**
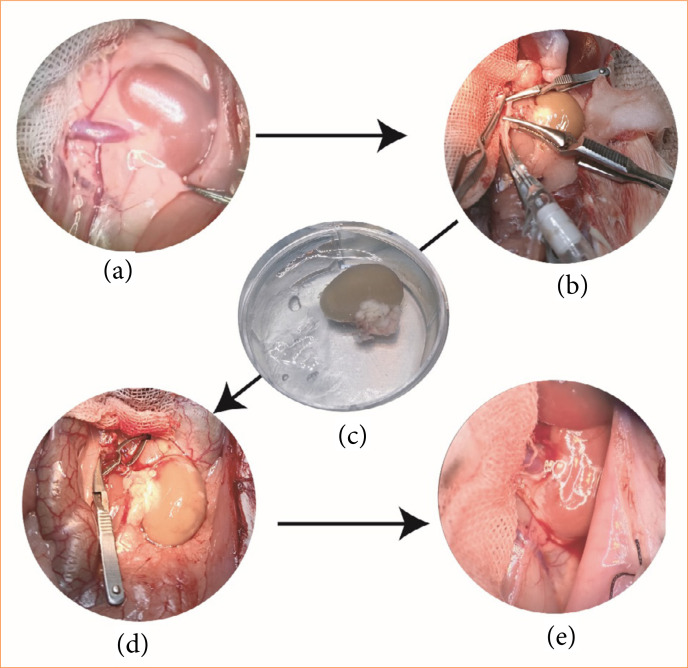
The protocol of a rat kidney transplantation model. Rats underwent surgery under general anesthesia. **(a)** After laparotomy, the intestine was placed on the right side of the abdomen to expose the left kidney and related blood vessels. **(b)** The left kidney was flushed *in situ* with cold organ preservation solution via a 22G venous catheter inserted into the lumbar aorta. **(c)** The harvested donor kidney was statistically stored in the same organ preservation solution. **(d)** After bilateral nephrectomy in recipient rats, the donor kidney was implanted *in situ* on the left renal bed, followed with end-to-end renal vessels anastomosis. **(e)** After the reconstruction of renal vessels and ureters, the kidney transplantation step was completed, and the donor kidney was reperfused.

Source: Elaborated by the authors.

A continuous suture was used for the end-to-end anastomosis of renal vein. Two hold-staying stitches with two 10/0-silk sutures were placed 180° apart. The dorsal walls of the veins were sutured continuously from the lumen between the two veins, and then the anterior walls were sutured similarly. Vein anastomosis was completed by continuous suturing with 10/0-silk suture. Thereafter, the artery clamp was released first to observe the kidney reperfusion, and then the clamp of renal vein was also released to complete the renal circulation. Ureter anastomosis was completed through suturing the donor ureter to recipient ureter by four–six interrupted sutures. All microvascular anastomoses were performed by a single microsurgeon (Fig. 1).

### Suturing techniques

#### Interrupted suture

**Figure 2:**
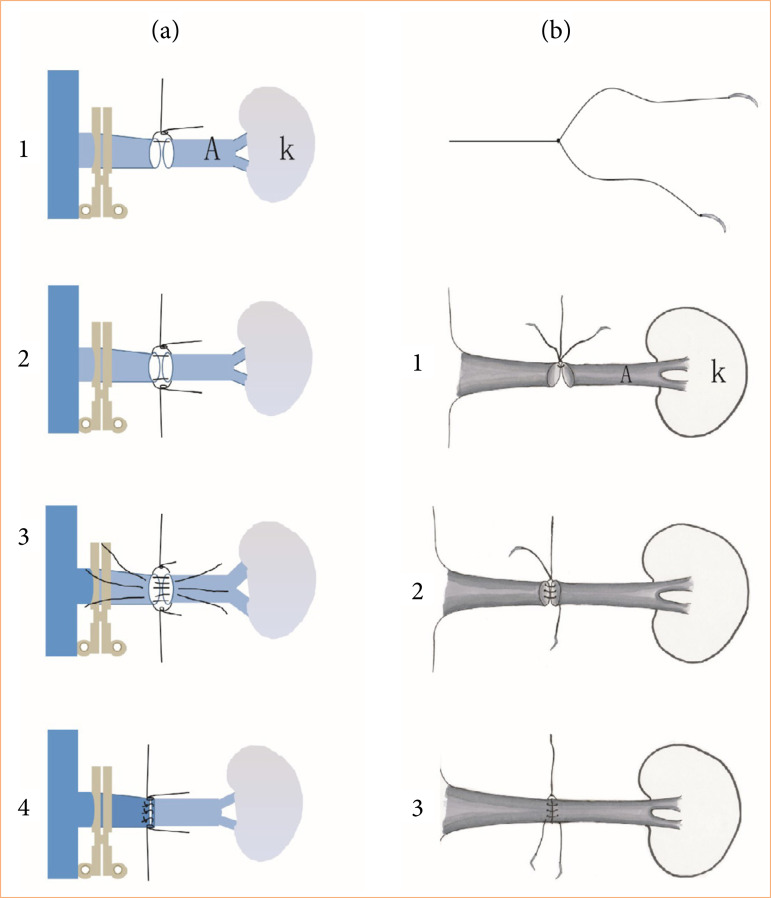
The schematic illustration of both the I suture technique and the Y-shape suture technique. **(a)** the schematic display of interrupted suturing technique. (1) After aligning the renal arteries of the donor and recipient rats, the interrupted suture of the upper corner was performed first, followed by suturing of the lower corner. (2) The lumens of the arteries were exposed clearly by pulling the up and down holding sutures. (3) Three interrupted stitches were intermittently sewn, first suture in the middle, then the upper stitch followed by the down stitch. (4) The interrupted sutures were tied and fixed in sequence. The posterior walls were also sutured in the same way. **(b)** The sketching displaying the Y-shape suture technique. Prepare a Y-shaped suture by tied the two sutures and cut off one tail suture, and so the Y suture poses a tail suture and two needle sutures. (1) The upper corner of the renal artery between the donor and recipient was firstly sutured, and the tail line of the Y-shaped suture was straightened to expose the artery lumens. (2) One needle of the Y-shaped suture to continuously suture was used to anastomose the posterior wall of the artery. The anterior walls of the arteries were exposed by straightening the already stitched thread by a vascular clamp. (3) The other needle of the Y-shaped suture was used to suture the anterior walls of the arteries and tie the last two threads in a knot to complete the artery anastomosis.

Two initial hold-staying sutures were placed at the left and right sides of the vessels. Both the anterior and posterior sides of the artery were completed using interrupted sutures (Fig. 2a).

A: the renal artery; K: kidney. Source: Elaborated by the authors.

#### Y-shape continuous suture technique

One hold-staying suture with the Y-shape suture bearing two needles was placed on the top corner of the arteries. The tail of the Y-shape suture was straightened with a small microvascular clamp to stretch the arteries slightly. Semicircular running sutures were performed on both the arterial ventral and dorsal walls. First, the arterial dorsal edges were anastomosed by applying three or four running stitches with the Y-shape suture. The needle was pierced out through the donor arterial wall and straightened with a clamp. Second, the arterial lumens were irrigated with saline to clear the view. Third, four equally spaced continuous sutures were placed on the ventral edges with the other needle of the Y-shape suture. Finally, the two ends were tied together (Fig. 2b).

#### Anterior-interrupted and posterior-continuous suture technique

**Figure 3:**
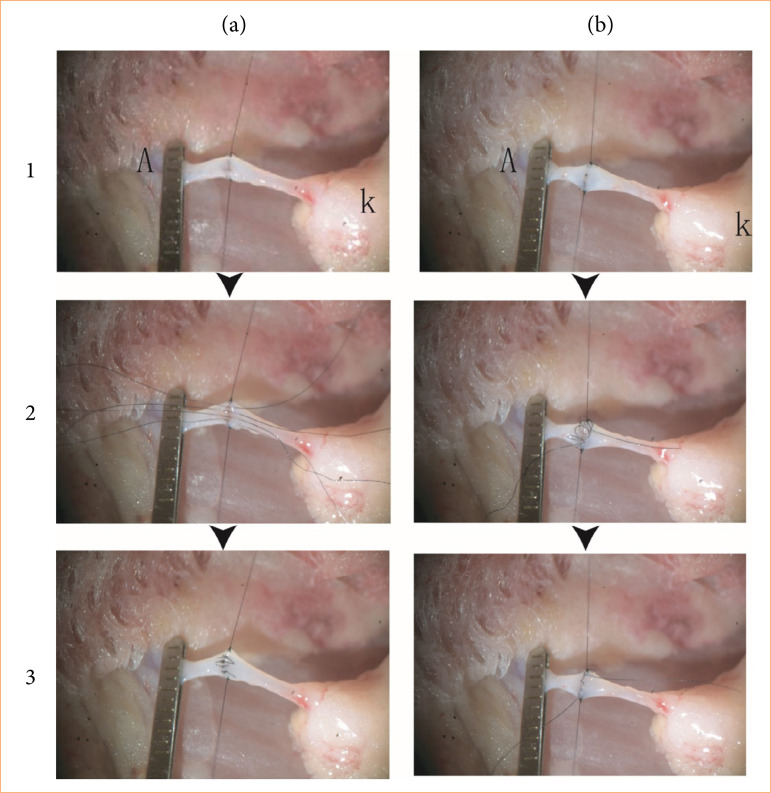
Protocol of the anterior-interrupted and posterior-continuous (I-C) suture technique. **(a)** Interrupted suture steps in the anterior wall of the renal artery. (1) Two holding sutures. (2) Interrupted sutures in the anterior wall. (3) Sutures finished with knots in the anterior wall. **(b)** Continuous suture steps in the posterior wall of the artery. (1) The posterior artery walls and vessel lumens were exposed clearly by pulling the up and down holding sutures. (2) The posterior walls of the donor and recipient arteries were continuously sutured, waiting for tightening once together. (3) The continuous suture is straightened to achieve complete vascular suturing.

Source: Elaborated by the authors.

Two hold-staying sutures were placed at the upper and lower corners of the arterial opening. The vessels were irrigated with saline to check its patency. Then, four stitches were placed in the anterior walls one by one in equal distance. Once all stitches were in place, the suture lines were knotted one by one, and the ends trimmed off (Fig. 3a). Then, both the donor and recipient renal arteries were similarly turned over 180° to expose the posterior walls.

The continuous sutures of this posterior wall were performed with the one stay suture, which were tied the other stay suture (Fig. 3b).

According to the suture fashion, animals were divided into three groups (n = 19, in each group):

- Interrupted suture group (I group);
- Y-shape continuous suture group (Y group);
- anterior-interrupted and posterior-continuous suture group (I-C group).

Buprenorphine (0.1 mg/kg BW/d) was administered subcutaneously for analgesia post-operation. The characteristics of artery anastomosis were assessed during the operation. The blood and arterial tissues were harvested on the seventh day after operation for further renal function and histopathological analysis.

### Measurements of anastomosis time and arterial patency

The anastomosis time was defined as the time of placement of the donor kidney into the abdomen until the complement of the end-to-end renal arterial suture. Arterial patency was checked after the removal of the micro-clamps, using the following indicators: recovery speed of the reperfused kidney, renal color, and arterial pulsation.

The quality of arterial suture was graded with scores ranging from 1 to 3 according to the volume of leaking blood and the degree of anastomotic stenosis (depending on the recovery time of kidney reperfusion):

- 1 score: bleeding more than 2 mL or severe stenosis (recovery more than 5 min);
- 2 score: bleeding less than 2 mL or moderate stenosis (recovery less than 3 min);
- 3 score: no bleeding or no stenosis (immediately recovery).

### Histopathology assessment

The patent specimens of the renal artery, 1 cm in length including the anastomotic site, were taken from each group for a histopathological assessment. Paraffin slides (4.0 μm) of arterial walls were sectioned and stained with hematoxylin-eosin (H-E) and Verhoeff’s van Gieson (EVG). The stained sections were assessed and scored for the damage degree according to the indicators in endothelial injury, proliferation, medial necrosis, the presence and location of the suture materials, and the degree of inflammatory reaction as previously reported^7^.

### Enzyme-linked immunosorbent assay

Whole blood was collected from the inferior vena cava and centrifuged at 3,000 rpm for 10 minutes. The supernatants were then transferred into two V-tubes and stored individually in a -80°C freezer for subsequent testing. Standard diagnostic kits from Kangte Biotechnology Co., in Zhejiang, China, were utilized to measure serum Cr and BUN levels.

### Statistical analysis

Data were expressed as mean (± standard deviation). After testing for normal distribution with the D’Agostino & Pearson omnibus normality test, analysis of variance or Kruskal-Wallis’ test was used to analyze anastomosis time. Fisher’s exact test or χ^2^ test was used to assess the significance of the patency rate and postoperative complication. Kaplan-Meier survival curves were used to show animal survival, and the long-rank test was used to determine statistical significance. Statistical significance level was set at *p* = 0.05.

## Results

### Operation parameters

An allogenic kidney transplantation model was utilized to analyze the effect of various suture techniques on the success rate of the model. The surgical procedures for the kidney transplants were consistent across the three groups, with similar body weights, kidney weights, and overall surgical duration (Table 1).

**Table 1:** Baseline parameters of the rat kidney transplantation (n = 19 in each of group).

	I Group	Y Group	I-C Group
Body weight (g)	289 ± 22	286 ± 17	287 ± 16
External diameter (mm)#	0.65 ± 0.17	0.67 ± 0.19	0.66 ± 0.18
Cold storage time (min)	95 ± 12	101 ± 12	97 ± 15
Warm ischemia time (min)	30.0 ± 3.4	27.7 ± 2.0	29.4 ± 2.8
Whole operation time (min)	117 ± 11	112 ± 13	113 ± 15

I group: interrupted suture; Y group: Y-shape continuous suture; I-C group: anterior-interrupted and posterior-continuous suture;

# measured in the renal arterial diameter during the implantation operation.

Source: Elaborated by the authors.

### Anastomosis characteristics and graft function assessments

The study revealed that the artery patency rate post-surgery was 100% across all three groups. The mean suture scores were notably low in the I group (1.35 ± 0.68) and the Y group (1.69 ± 0.82), but significantly increased in the I-C group (2.21 ± 0.71, *p* < 0.05), indicating an enhancement in suture quality with the I-C technique. Moreover, the time required for anastomosis was shorter in the Y (16.35 ± 2.38) and I-C (18.85 ± 1.61) groups compared to the I group (22.51 ± 3.11, *p* < 0.05). Notably, graft function recovery was higher in the I-C suture group, as evidenced by lower serum creatinine levels on day 7 post-transplantation (I-C group: 44.69 ± 32.99 vs. I group: 129.69 ± 115.65, *p* = 0.04; and vs. Y group: 95.38 ± 76.80 mg/dL, *p* = 0.41). Consequently, the seven-day survival rate of renal grafts was superior in the I-C suture group (90%) compared to the I and Y groups (75 and 60%, respectively), underscoring the enhanced success of the transplantation model with the I-C suture technique. Rats that expired within seven days post-surgery were identified through abdominal cavity exploration to determine the primary cause, mainly postoperative anastomotic bleeding or stenosis.

### Histopathology of vessels

**Figure 4:**
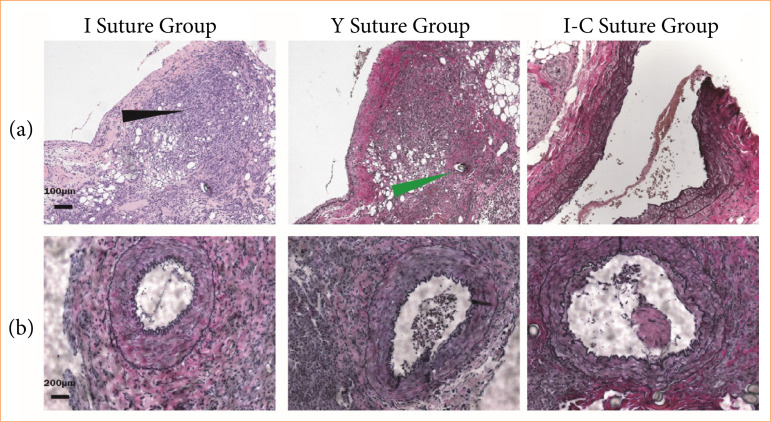
The histopathological characteristics of arterial walls on day 7 post-operationally. **(a)** The images of artery hematoxylin-eosin staining. Histopathological changes of the vascular wall at the arterial anastomotic site show thickening of connective tissue (black arrow), in which the residual suture is visible (green arrow). **(b)** Representative images of Verhoeff’s van Gieson stain of the arterial wall among the three groups. The elastic fiber layer (black wavy linear) and the red collagen fiber layer within the vascular wall are clear in the three groups. A small amount of thrombus can be seen in the endothelial layer of the lumen stained with blood vessels in the I suture group.

Source: Elaborated by the authors.

Postoperative features of vessel walls were confirmed using H-E and EVG stains. H-E stains revealed the layers of vessel walls and the position of sutures, indicating that the vessel suture was full-thickness (Fig. 4a). Elastic and collagen fibers in the vascular wall showed significant recovery (Fig. 4b). Histopathological changes were scored and analyzed across the three groups. Table 2 demonstrated that the I-C suture technique resulted in significantly more damage to vascular endothelial and muscle tissues compared to the I suture technique, along with a reduced inflammatory response (Table 2).

**Table 2:** Pathological scores of sutured arteries on day 7 after anastomosis.

Vascular changes	I suture group	Y suture group	I-C suture group
Endothelial injury	5	4	3*
Endothelial proliferation	3	1	2
Muscles damage	4	3	2*
Inflammatory reaction	4	3	2*
Suture materials degree	2	3	2

I group: interrupted suture; Y group: Y-shape continuous suture; I-C group: anterior-interrupted and posterior-continuous suture;

* *p* < 0.05, compared to I suture group.

Source: Elaborated by the authors.

## Discussion

In this study, our research team successfully established a reliable rat model for orthotopic kidney transplantation between Fisher-Lewis rats to study chronic allograft injury. The *in-situ* transplantation of kidneys and the end-to-end anastomosis of corresponding renal arteries are widely accepted as the standard method for rat kidney transplantation^8^. The quality of arterial anastomosis is crucial for the stability of rat kidney transplantation models.

Our findings indicate that both the Y-shape technique and the anterior-interrupted and posterior-continuous (I-C) suture technique are efficient and timesaving compared to the traditional interrupted suture technique. This was supported by the reduced artery suturing time, immediate suture quality, and improved graft survival post vascular anastomosis. Both the Y-shaped suture technique and the I-C suture technique have significantly reduced anastomosis times compared to the conventional interrupted suture method. The I-C suture technique demonstrated the highest rate of seven-day survival in the grafted animals among the three groups.

Continuous suture technique has been shown to shorten the anastomosis time and achieve a similar opening rate as intermittent suture^9^. However, there are concerns regarding the pocket effect and risk of anastomotic stenosis associated with continuous suturing. Various technical modifications have been reported by different microsurgeons. For example, Hamilton et al.^10^ introduced a circumferential continuous suture technique with a single-stop suture, resulting in a 92.6% patency rate in rabbit femoral arteries. Simsek et al.^11^ utilized a continuous horizontal mattress suture technique in rat carotid artery anastomosis, leading to a 90% patency rate and reduced anastomosis time. Cigna et al.^12^ described the posterior wall first continuous interrupted airborne technique, which is used in current renal vein anastomosis.

The Y-shape technique was developed to avoid arterial wall turnover and provide a clear operative field for microvascular anastomosis, aiming to minimize operation time and enhance procedural security. Previous studies have also suggested alternative techniques, such as placing sutures in the posterior wall of the vessel to improve visibility during anastomosis^13^. Our data showed that the Y-shape suture technique reduced anastomosis time and vascular bleeding, although thrombotic occlusions were observed, possibly due to the purse-string effect of continuous suture causing slow blood flow within the vascular lumen.

The interrupted suture technique is commonly used in micro-arterial anastomosis, despite drawbacks such as posterior wall catching, multiple knots, and time consumption. Various modifications have been proposed to address these challenges. Chen et al.^14^ introduced the spiral interrupted suture technique, combining continuous suturing with interrupted ties to enhance efficiency and security. Ulusal et al.^15^ previously demonstrated a combined technique using continuous sutures and interrupted ties, but faced issues with short thread length for knot separation. Watanabe et al.^16^ reported a combined suture method involving continuous suturing on the posterior wall and intermittent suturing on the anterior wall, resembling the I-C technique in this study. However, the posterior wall first suturing technique poses a risk of arterial intima inversion and thrombosis due to continuous suturing within the vascular lumen.

Considering previous suture techniques and rat renal artery characteristics, the I-C suture technique was developed for small artery suturing. This technique allows vessel dilatation and reduces anastomosis time. Additionally, our research group utilized the posterior wall first suture technique for renal vein anastomosis to expedite suturing, given the large venous lumen and thin vascular wall of the renal vein.

To enhance the diameter of anastomotic blood vessels, some suggest utilizing end-to-side anastomosis techniques^17^^,^ 18. While these techniques achieve similar speed to end-to-end anastomosis, they require blocking the abdominal aorta, which can disrupt lower body blood circulation in rats and impede postoperative recovery. Various arterial anastomosis methods, such as cuff, stent, and sleeve techniques, are employed to improve the success of rat kidney transplantation models^19^^,^ 20. However, these approaches carry a heightened risk of luminal thrombosis. The use of foreign body-assisted vascular anastomosis may negatively impact the long-term function recovery of transplanted kidneys after surgery. Therefore, it is recommended to avoid these techniques in models of chronic transplant rejection. An effective technique should prevent vessel stenosis and thrombosis at the anastomosis site. Two key principles are suggested for small artery anastomosis: first, the use of eversion sutures to maintain a smooth internal lumen, and second, the adoption of partially interrupted sutures to allow for arterial dilation postoperatively^21^.

## Conclusion

Our study demonstrated that anterior-interrupted and posterior-continuous suture method is superior to the interrupted suturing technique. Therefore, we suggest the anterior-interrupted and posterior-continuous suture technique as an interesting alternative for vascular anastomosis of the rat kidney transplant model.

## Data Availability

The datasets used and/or analyzed during the current study are available from the corresponding author on reasonable request.
